# Atomic disorder of Li_0.5_Ni_0.5_O thin films caused by Li doping: estimation from X-ray Debye–Waller factors

**DOI:** 10.1107/S1600576715020002

**Published:** 2015-11-19

**Authors:** Anli Yang, Osami Sakata, Ryosuke Yamauchi, L. S. R. Kumara, Chulho Song, Yoshio Katsuya, Akifumi Matsuda, Mamoru Yoshimoto

**Affiliations:** a Synchrotron X-ray Station at SPring-8, National Institute for Materials Science (NIMS),1-1-1 Kouto, Sayo, Hyogo 679-5148, Japan; b Synchrotron X-ray Group, Quantum Beam Unit, NIMS, 1-1-1 Kouto, Sayo, Hyogo 679-5148, Japan; c Department of Innovative and Engineered Materials, Tokyo Institute of Technology, 4259-J3-16 Nagatsuta-cho, Midori-ku, Yokohama, Kanagawa 226-8502, Japan; d Global Research Center for Environment and Energy Based Nanomaterials Science, Lithium Air Battery Specially Promoted Research Team, NIMS, 1-1 Namiki, Tsukuba, Ibaraki 305-0044, Japan

**Keywords:** atomic disorder, thin films, Li doping, Debye–Waller factors

## Abstract

Cubic type room-temperature (RT) epitaxial Li_0.5_Ni_0.5_O and NiO thin films with [111] orientation grown on ultra-smooth sapphire (0001) substrates were examined using synchrotron-based thin-film X-ray diffraction. The 11 and 22 rocking curves including six respective equivalent reflections of the Li_0.5_Ni_0.5_O and NiO thin films were recorded. The RT *B*_1_ factor, which appears in the Debye–Waller factor, of a cubic Li_0.5_Ni_0.5_O thin film was estimated to be 1.8 (4) Å^2^ from its 11 and 22 reflections, even though the Debye model was originally derived on the basis of one cubic element. The corresponding Debye temperature is 281 (39) K. Furthermore, the *B*_2_ factor in the pseudo-Debye–Waller factor is proposed. This parameter, which is evaluated using one reflection, was also determined for the Li_0.5_Ni_0.5_O thin film by treating Li_0.5_Ni_0.5_O and NiO as ideal NaCl crystal structures. A structural parameter for the atomic disorder is introduced and evaluated. This parameter includes the combined effects of thermal vibration, interstitial atoms and defects caused by Li doping using the two Debye–Waller factors.

## Abstract 2

The structures of room-temperature epitaxial Li_0.5_Ni_0.5_O and NiO thin films are compared. A structural parameter for the atomic disorder is discussed and evaluated using X-ray Debye–Waller factors.

## 1. Introduction   

Li
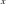
Ni
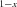
O has attracted considerable attention recently as a potential material for high-performance electrochromic devices (Moulki *et al.*, 2012 ▸), UV detectors (Ohta *et al.*, 2003 ▸) and gas sensors (Garduno-Wilches & Alonso, 2013 ▸). Recently, cubic type NiO thin films with (111) orientation containing large amounts of Li (up to 50 mol%) were epitaxically grown on ultra-smooth sapphire (0001) substrates by room-temperature (RT) pulsed laser deposition (Shiraishi *et al.*, 2010 ▸; Yang *et al.*, 2014 ▸). The ultra-smooth sapphire substrate used in this work has an atomic ‘step and terrace’ structure on its surface, which consists of atomically flat terraces comprising single oxygen layers separated by periodic atomic steps with 0.22 nm height corresponding to the oxygen layer spacing along the *c* axis. These films could be grown even though bulk NiO containing over 30 mol% Li undergoes a phase transformation from the cubic to rhombohedral structure (Goodenough *et al.*, 1958 ▸).

The Debye temperature is an important characteristic structural parameter of a solid and describes the dynamic motions of atoms in the material. A number of physical parameters such as mean-square atomic displacement (Herbstein, 1961 ▸) and elastic constant (Gazzara & Middleton, 1964 ▸) depend on the Debye temperature of a solid. Therefore, it is very important to determine the Debye temperature of Li
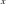
Ni
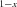
O in order to understand its physical properties, such as the change of heat capacity caused by Li doping, and develop related electronic devices. X-ray diffraction is a valuable method to study thin films and other epitaxial layer materials. The changes of the lattice constant and crystal quality caused by doping are frequently evaluated using this method (Dutta *et al.*, 2010 ▸; Palatnikov *et al.*, 2006 ▸). However, the effect of atomic disorder caused by doping elements on the X-ray diffraction intensity has seldom been estimated (Sakata & Hashizume, 1997 ▸). In this study, we attempted to quantitatively analyze the effect of atomic disorder caused by Li doping on the X-ray diffraction intensity of an Li
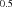
Ni
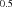
O thin film.

## 2. History of Debye temperature estimation by X-ray diffraction   

Originally, the Debye temperature was determined using X-ray diffraction and was strictly limited to monoatomic cubic crystals. The integrated intensity, *I*, from a cubic sample can be expressed as (James, 1962 ▸) *I* = 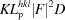, where *K* is a constant, which is a normalized factor depending on the experimental arrangement, 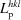 is the Lorentz–polarization factor 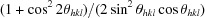, *h*, *k* and *l* are the Miller indices of the diffraction plane, θ is the Bragg diffraction angle, 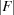 is the modulus of the structure factor, and *D* is the Debye–Waller factor, expressed as 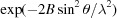. Here (James, 1962 ▸) 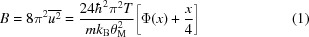
and 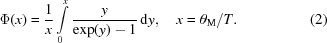


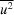
 is the mean-square displacement of atoms from the sites of the average lattice of the solid solution. *m* is the mass of the atom, 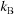 is the Boltzmann constant, 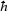 is Planck’s constant divided by 2π, 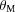 is the Debye temperature and *T* is the experimental temperature.

Later, the Debye theory was extended to apply to binary alloys, such as KCl and NaCl, whose specific heats can be fairly well described by the Debye–Waller formula with an appropriate value of the characteristic temperature (James, 1962 ▸). The structure factor for binary alloys is formally written as (Murakami, 1953 ▸) 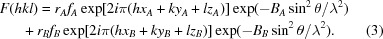
Here *f* is the atomic scattering factor, *x*, *y*, *z* are the atomic positions in the unit cell, and the subscripts *A* and *B* refer to the two components of the alloy. 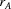 and 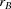 are the atomic fractions of *A* and *B*, respectively. To perform a simple approximation (Kulkarni & Bichile, 1977 ▸; Wathore & Kulkarni, 1980 ▸; Pathak & Trivedi, 1973 ▸), we let 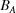 = 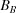 = 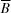, and in equation (1) use 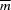 = 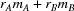. This approximation allows a quasi-Debye temperature to be obtained. The experimental results summarized by Lonsdale (1948 ▸) are in reasonable agreement with this approximation.

## 3. Introduction of a structure parameter   

**Figure 1:**
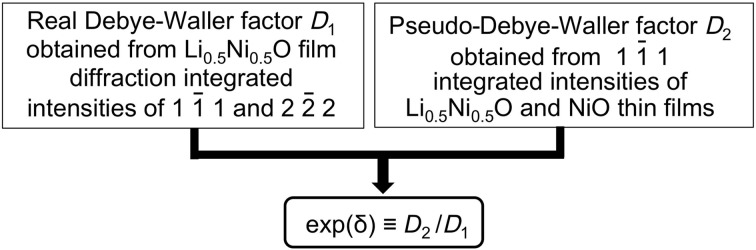
The definition of a structure parameter [exp(δ)] to describe the effect of atomic disorder caused by Li doping on the X-ray diffraction intensity of an Li
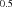
Ni
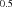
O thin film.

The Debye temperature has been evaluated from X-ray intensities not only at different temperatures for a Bragg peak (Herbstein, 1961 ▸) but also from different reflections at a given temperature (Herbstein, 1961 ▸; Kulkarni & Bichile, 1977 ▸). Here, a real Debye–Waller factor 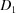 for an Li
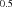
Ni
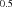
O thin film was obtained by X-ray diffraction using two different reflections, 1
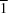
1 and 2
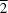
2, with the assumption that the rock-salt lattice is a simple cubic structure composed of one kind of atom and the Li atoms occupy Ni substitutional positions without any atomic disorder. We also assume that the thermal disorder contributes to the ratio of the diffracted intensities between the 1
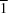
1 and 2
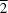
2 reflections for simplicity. Second, a pseudo-Debye–Waller factor 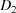 for the Li
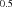
Ni
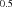
O thin film was obtained from the X-ray 1
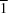
1 intensity ratio between Li
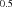
Ni
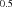
O and NiO. Finally, we introduce an atomic disorder parameter exp(δ) (δ 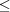 0), including the combined effects of thermal vibration, interstitial atoms and defects expressed using 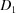, 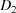 and the reported NiO Debye temperature (Freer, 1981 ▸). This term is required because the positions of atoms are changed by Li doping. This process is outlined in Fig. 1 ▸.

## 4. Samples and experiment   

Li
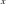
Ni
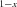
O thin films were epitaxically grown on ultra-smooth sapphire (0001) substrates (Yoshimoto *et al.*, 1995 ▸) by RT pulsed laser deposition. Details of the deposition conditions have been reported elsewhere (Shiraishi *et al.*, 2010 ▸; Yang *et al.*, 2014 ▸). The compositions of NiO thin films containing large amounts of Li grown at RT were determined using inductively coupled plasma atomic emission spectrometry (Shimadzu ICPS-8100). The lithium contents *x* in the Li
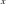
Ni
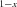
O samples were 0 and 0.5. The periodicities of the intensity oscillations appearing in the X-ray reflectivity curves revealed that the thicknesses *t* of the NiO and Li
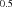
Ni
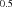
O epitaxial thin films were 400 (21) and 231 (6) Å, respectively.

X-ray diffraction measurements were performed with a six-axis diffractometer at the National Institute for Materials Science (NIMS) beamline BL15XU, SPring-8. The wavelength used was 1.000 Å.

## 5. Results and discussion   

### 5.1. Crystal structure   

The crystallographic epitaxy between the Li
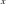
Ni
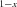
O thin films and sapphire substrate was Li
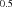
Ni
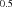
O [1
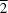
1] // sapphire [11
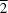
0] and Li
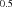
Ni
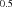
O [111] // sapphire [0001]. The cube-on-hexagonal epitaxial relationship was the same as that reported previously for similar samples (Sakata *et al.*, 2004 ▸; Yang *et al.*, 2014 ▸). The φ scan around the nonspecular NiO (1
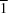
1) Bragg positions showed sixfold symmetry (not shown here). This is because two types of terraces formed alternately on the sapphire (0001) face, with 0.2 nm-high steps (Yamauchi *et al.*, 2012 ▸). We also recorded the X-ray intensities of six 1
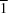
1 and 2
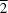
2 diffraction peaks.

### 5.2. Debye temperature of an Li_0.5_Ni_0.5_O epitaxial thin film   

**Figure 2:**
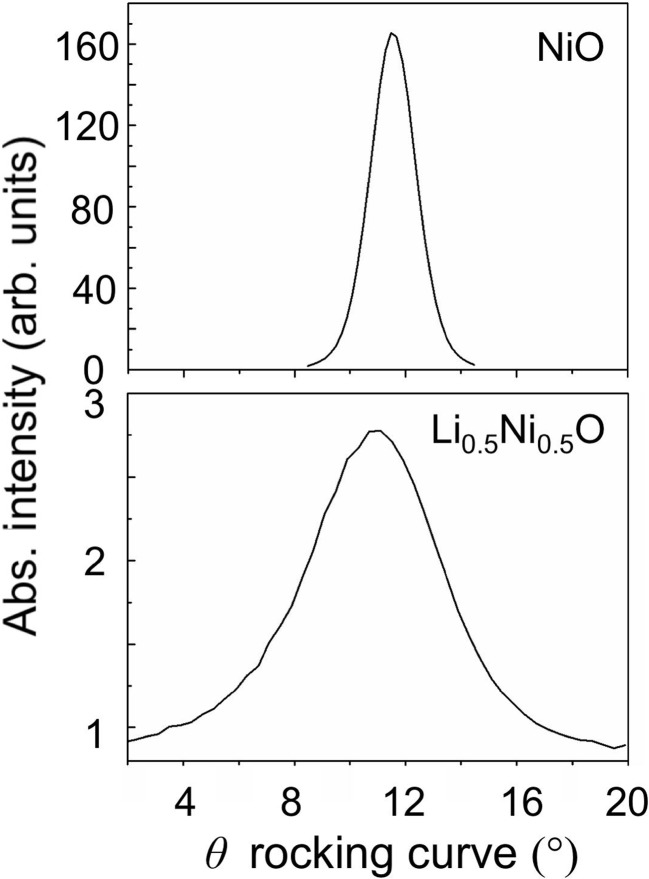
X-ray rocking curves around the 1
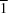
1 Bragg peaks of NiO and Li
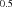
Ni
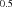
O thin films.

Fig. 2 ▸ shows one of the six X-ray rocking curves around the 1
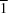
1 Bragg peaks of the NiO and Li
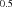
Ni
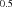
O thin films. The average full width at half-maximum of the Li
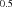
Ni
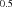
O thin film (5.48°) was much larger than that of the NiO thin film (1.60°). This suggests that the crystal quality was lowered after Li doping.

The average integrated intensity ratio 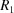 between the 1
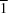
1 and 2
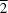
2 Bragg peaks of the Li
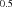
Ni
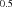
O epitaxial thin film was calculated to be 2.5 (2). The contribution of thermal diffuse scattering (TDS) to the measured intensity was neglected in our analysis, because the TDS correction was small (less than 4% even in imperfect single-crystal silicon; Matsumuro *et al.*, 1990 ▸; Chipman & Batterman, 1963 ▸) in our samples compared with the statistical fluctuation, which was about 7.6% in the NiO thin film. If we assume that all Li atoms are substituted at Ni atom positions, the composition of the Li
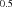
Ni
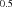
O epitaxial thin film is uniform and structural disorder is neglected, the diffracted intensity from the Li
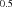
Ni
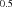
O epitaxial film is 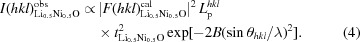



Let us introduce the ratio 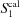 of the square of the calculated structure factors: 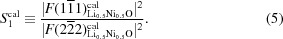
The values of 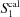 are estimated to be 0.331 and 0.328 when we assume that the NiO and Li
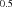
Ni
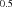
O films have the NaCl crystal structure and use the atomic scattering factors (Brown *et al.*, 2006 ▸) for their neutral atoms (Li, Ni and O) and ions (Li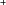, Ni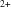 and O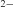), respectively. The calculated results were almost the same. Let us define the observed integrated intensity ratio 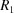: 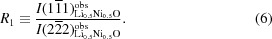
The averaged value of 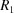 for the six equivalent reflections was 2.5 (2). Substituting equation (4) into equation (6) allows us to derive the formula for 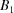 as follows: 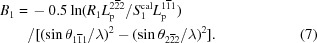
The 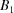 factor of Li
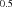
Ni
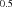
O was calculated to be 1.8 (4) Å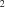 when we used parameters observed from the Li
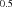
Ni
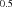
O films: 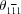 = 12.19° and 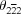 = 24.88°, and (1
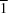
1) lattice spacing = 2.368 (4) Å. The corresponding Debye temperature was 281 (39) K according to equations (1) and (2). The error originates from that of 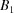, and it is a little larger than that already determined by X-ray diffraction (Herbstein, 1961 ▸), so it may be caused by the large statistical fluctuation of integrated intensity. The corresponding r.m.s. amplitude of atomic vibration was evaluated to be 0.15 Å, as shown in Table 1 ▸. The ratio *r* between the r.m.s. amplitude of atomic vibration and the (1
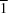
1) lattice spacing of the Li
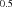
Ni
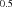
O thin film was 6.3%. This value is larger than that of ideal bulk NiO (4.6%; Freer, 1981 ▸), which suggests that Li doping increased the lattice thermal vibration (Borca *et al.*, 2000 ▸). 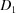 is written as 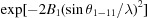 and is here called the real Debye–Waller factor for the Li
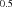
Ni
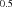
0 thin film.

### 5.3. Estimation of the effect of atomic disorder caused by Li doping on the X-ray diffraction intensity   

**Figure 3:**
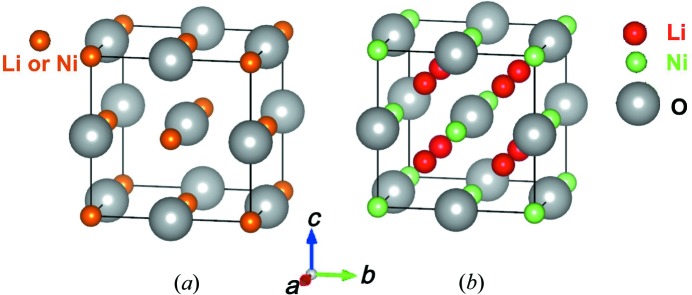
Crystal structure models of Li
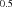
Ni
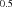
O with (*a*) all of the Li atoms substituted for Ni atoms and (*b*) all of the Li atoms located in interstitial sites.

In order to obtain the structure parameter that can be used to estimate the effect of atomic disorder [exp(δ)] caused by Li doping on the X-ray diffraction intensity of an Li
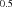
Ni
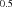
O thin film, let us propose a factor 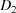 from the X-ray 1
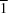
1 intensity ratio between Li
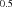
Ni
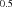
O and NiO as shown in Fig. 1 ▸. If we assume that all of the Li atoms are substituted for Ni atoms and the composition of the Li
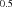
Ni
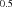
O epitaxial thin film is uniform (Fig. 3 ▸ *a*), then structural disorder can be neglected.

Let us define the measured average intensity ratio 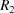 between the 1
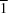
1 Bragg peaks of the Li
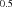
Ni
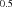
O and NiO films:
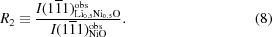
The average value of 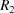 for the six equivalent reflections was 0.029 (4). We also define
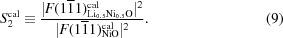
The value of 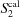 is 0.148 when we assume that the NiO and Li
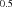
Ni
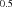
O films have the NaCl crystal structure and use the atomic scattering factors for their neutral atoms. The formula for our proposed parameter 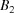 is obtained after we substitute equation (4) into equation (8): 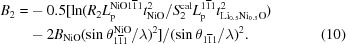


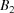
 was evaluated to be 6.5 (15) Å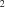 using the 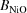 value of 0.939 Å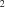 obtained from 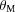 (317.4 K at RT; Freer, 1981 ▸) of NiO according to equations (1) and (2). We also used parameters (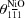 = 12.05°) observed from the NiO film. These obtained parameters, the r.m.s. amplitude of atomic vibration and its ratio compared with the lattice spacing are listed in Table 2 ▸. 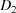 is written as 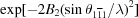 and is here called the pseudo-Debye–Waller factor for the Li
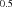
Ni
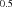
0 thin film. 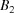 is much greater than 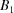. We do not consider the atomic disorder when obtaining the 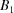 value. To consider the difference between 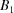 and 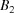, let us assume the relation of 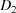 and 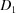 using the atomic disorder parameter expressed as 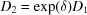. 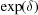 was evaluated to be 0.66 using NiO as reference. This value is much smaller than unity, which suggests that the range in which the atoms are located might be *ca* 0.14 Å wider than that for 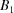. In general, Debye–Waller factors may include not only the influence of the thermal disorder but also that of the atomic disorder. The structural disorder that we evaluated looks to have a very small influence on 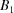. Furthermore, let us assume another extreme case where all of the Li atoms are located on interstitial sites. Interstitial Li atoms are found not only in cubic Li-doped NiO (Guo *et al.*, 2013 ▸) but also in many other crystal structures like ZnO (Chawla *et al.*, 2009 ▸), ZnSe (Sasaki *et al.*, 1993 ▸) and Li
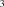
NbO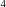 (McLaren *et al.*, 2004 ▸). The positions of the Li atoms are (1/4, 1/4, 1/4), (3/4, 1/4, 1/4), (3/4, 3/4, 1/4), (1/4, 3/4, 1/4), (1/4, 1/4, 1/2), (3/4, 1/4, 1/2), (3/4, 3/4, 1/2), (1/4, 3/4, 1/2), as shown in Fig. 3 ▸(*b*). Therefore, the structure factor of Li
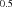
Ni
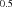
O along [1
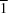
1] changes to 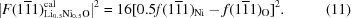
Let us introduce 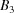 for all the Li atoms sitting in interstitial sites. The value is 3.1 (15) Å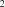 using equations (8) and (11), as shown in Table 2 ▸. The difference between 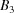 for the Li interstitial model and 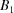 is smaller than that between 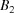 for the Li substitute model and 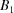. This implies that some Li atoms are probably located in the interstitial sites in a heavily doped Li
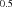
Ni
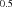
O thin film. These results help us to understand the physical properties of Li
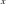
Ni
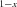
O to develop Li
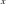
Ni
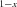
O-based devices.

## 6. Concluding remarks   

In conclusion, the conventional 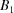 factor of an Li
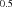
Ni
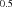
O thin film was estimated to be 1.8 (4) Å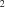 using X-ray diffraction by considering the 1
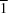
1 and 2
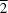
2 reflections. The corresponding Debye temperature was 281 (39) K. Furthermore, the pseudo-Debye–Waller factor of the Li
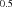
Ni
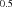
O thin film was obtained using the intensity ratio between the 1
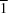
1 Bragg peaks of Li
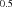
Ni
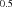
O and NiO thin films. The atomic disorder parameter that we proposed was evaluated to be 0.66. The disorder may include combined effects of thermal vibration, interstitial atoms and defects caused by Li doping. Interstitial Li atoms could be present in heavily Li-doped Li
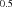
Ni
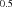
O thin films because the proposed 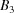 factor was smaller than 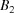 determined for a film with Li ions only in Ni sites.

## Figures and Tables

**Table 1:** *B*_1_ and *B*_NiO_ factors, Debye temperature θ_M_, r.m.s. amplitude of atomic vibration 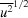, (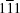) lattice spacing 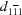, and ratio 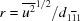 of Li_0.5_Ni_0.5_O and NiO thin films at 298 K

Sample	*B*_1_ or *B*_NiO_ (Å^2^)	θ_M_ (K)	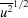 (Å)	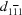 (Å)	*r* (%)
Li 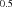 Ni 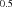 O	1.8 (4)	281 (39)	0.15 (2)	2.368 (4)	6.3 (8)
NiO	0.939	317.4 (Freer, 1981 ▸)	0.11	2.395 (2)	4.6

**Table 2:** *B*_2_ and *B*_3_ factors, Debye temperature θ_M_, r.m.s. amplitude of atomic vibration (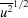), (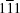) lattice spacing 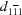, and ratio 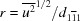 of Li_0.5_Ni_0.5_O thin films at 298 K

Site	*B*_2_ or *B*_3_ (Å 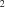 )	θ_M_ (K)	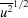 (Å)	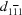 (Å)	*r* (%)
Substitutional	6.5 (15)	147 (22)	0.29 (3)	2.368 (4)	12.2 (13)
Interstitial	3.1 (15)	213 (45)	0.20 (6)	2.368 (4)	8.4 (25)
